# The Impact of Self Compassion on Eating Pathology: A Systematic Review Across Clinical and Community Samples

**DOI:** 10.1192/j.eurpsy.2026.11380

**Published:** 2026-07-31

**Authors:** B. Çam, S. T. Tabur, S. Bektaş

**Affiliations:** 1 Institute of Psychiatry, Psychology and Neuroscience, King’s College London, London, United Kingdom; 2 Department of Clinical Psychology, Hasan Kalyoncu University, Gaziantep, Türkiye

## Abstract

**Introduction:**

Self-compassion has emerged as a robust protective factor against disordered eating, consistently linked to lower levels of body dissatisfaction, shame, and emotional dysregulation. This systematic review critically examines the evidence on self-compassion’s role in eating psychopathology and evaluates the therapeutic potential of compassion-based interventions across clinical and community populations.

**Objectives:**

This review aimed to (1) synthesise evidence regarding the relationship between self-compassion and disordered eating symptoms, (2) examine the contribution of fear of self-compassion as a clinically relevant vulnerability factor, and (3) assess the reported outcomes of compassion-focused interventions designed to target eating-related psychopathology.

**Methods:**

A systematic literature review was conducted using PubMed, PsycINFO, and Scopus databases to identify peer-reviewed articles published between 2007 and 2024. Search terms included combinations of “self-compassion,” “eating disorders,” “body image,” “binge eating,” and “shame.” Eligible studies employed validated measures of self-compassion, reported quantitative findings, included clinical or non-clinical samples, and received appropriate ethical approval. Narrative synthesis was applied to integrate and interpret the results.

**Results:**

A consistent pattern of findings across included studies indicated that higher self-compassion is associated with reduced severity of disordered eating symptoms, including lower levels of shame, emotional eating, and negative body image. Fear of self-compassion was identified as a notable barrier to treatment, particularly in clinical samples, where it was associated with increased self-criticism and emotional avoidance. Compassion-based interventions such as Compassion-Focused Therapy and structured self-compassion exercises were found to improve emotion regulation and reduce eating pathology when delivered across multiple sessions, both online and in-person.

**Conclusions:**

Self-compassion has been demonstrated to function as a protective and therapeutic factor in the context of eating-related psychopathology. Its integration into evidence-based treatments may enhance emotional resilience, reduce maladaptive self-criticism, and support sustained recovery. Further high-quality, cross-cultural randomised controlled trials are warranted to evaluate long-term outcomes and underlying mechanisms of change.

**Disclosure of Interest:**

None Declared

